# Drug responsiveness of leukemic cells detected in vitro at diagnosis correlates with therapy response and survival in patients with acute myeloid leukemia

**DOI:** 10.1002/cnr2.1362

**Published:** 2021-03-06

**Authors:** Maria A. Kolesnikova, Aleksandra V. Sen'kova, Tatiana I. Pospelova, Marina A. Zenkova

**Affiliations:** ^1^ Institute of Chemical Biology and Fundamental Medicine SB RAS Novosibirsk Russia; ^2^ Novosibirsk Hematology Center Novosibirsk Russia; ^3^ Novosibirsk State Medical University Novosibirsk Russia

**Keywords:** acute myeloid leukemia, chemotherapy, drug responsiveness, survival

## Abstract

**Background:**

Acute myeloid leukemia (AML) is the most common acute leukemia in adults, and chemotherapy remains the most commonly used treatment approach for this group of hematological disorders. Drug resistance is one of the predictors of unfavorable prognosis for leukemia patients.

**Aim:**

The purpose of this study was to perform a retrospective analysis of the survival rate in AML patients according to age, tumor status, and chemotherapy regimen received and to analyze the therapy response of AML patients depending on the treatment received, initial responsiveness of tumor cells to chemotherapeutic drugs measured in vitro at diagnosis and expression of immunological markers.

**Methods:**

The survival of AML patients (*n* = 127) was analyzed using the Kaplan‐Meier method. Drug sensitivity of tumor cells of AML patients (*n* = 37) and the expression of immunological markers were evaluated by the WST test and flow cytometry, respectively. Correlation analysis was performed using Spearman's rank order correlation coefficient.

**Results:**

We found the treatment regimen to be the defining factor in the patient survival rate. In addition, the initial responsiveness of tumor cells to chemotherapeutic drugs measured in vitro at diagnosis correlated with the therapy response of AML: patients with high tumor cell sensitivity to particular cytotoxic drugs demonstrated a good response to treatment including these drugs, and patients with initial resistance of tumor cells to a particular chemotherapeutic agents and received it according to the clinical protocols demonstrated a poor response to antitumor therapy. Correlations of drug resistance in leukemic cells with the expression of immature and aberrant immunophenotype markers as established unfavorable prognostic factors confirm our assumption.

**Conclusion:**

The evaluation of the responsiveness of tumor cells to chemotherapy in vitro at diagnosis can be a useful tool for predicting the response of leukemia patients to planned chemotherapy.

## 1 INTRODUCTION

Acute myeloid leukemia (AML) is the most common acute leukemia in adults, responsible for approximately 150 000 deaths per year worldwide.^1^, ^2^ The main treatment approach for AML remains chemotherapy, which can be either inductive or palliative.^3^, ^4^ The general therapeutic strategy for AML patients has not changed substantially in more than 30 years. Nowadays, initial assessment serves to consider whether the patient is eligible for intensive induction chemotherapy.^4^, ^5^ If induction treatment is started as soon as possible in accordance with standard protocols, a better response to therapy is achieved.^6^

Assignment of the cytotoxic treatment and prediction of the therapy response for AML patients depends on patient‐associated factors (age, coexisting conditions, poor performance status) and disease‐related factors (white‐cell count, prior myelodysplastic syndrome or cytotoxic therapy, leukemic‐cell genetic changes).^5^ However, the most serious obstacle to effective specific treatment of leukemia is still the resistance of leukemic cells to cytotoxic drugs, which not only forms as the result of chemotherapy, but also arises spontaneously as an individual tumor characteristic in patients who have not previously received antitumor therapy.^7^ An initial assessment of the drug sensitivity/resistance of tumor cells, as well as introduction of methods providing for rapid and reliable prediction of patients' response to the planned chemotherapy are urgently needed in clinical practice.^8^

Previously, we have shown that the drug responsiveness of tumor cells detected in vitro at diagnosis correlates with the therapy response and established prognostic markers in leukemia patients.^9^ The most significant correlations were identified for AML: patients with tumor cell resistance to chemotherapeutic drugs demonstrated a poor response to standard chemotherapy and carried immature and aberrant immunological markers and cytogenetic abnormalities in tumor cells associated with an unfavorable prognosis for AML patients.^9^ The aim of the present study was to prove our conception that an evaluation of the initial responsiveness of tumor cells to chemotherapeutic drugs in vitro can be useful in predicting the therapy response of leukemia patients and to assign the most effective antitumor treatment.

## 2 MATERIALS AND METHODS

### 2.1 Patients

The study included 127 patients with AML. All patients were enrolled in the study by signing the informed consent and with ethical approval from the Institutional Review Board of Novosibirsk State Medical University (No. 80/2015) in accordance with the ethical principles of the Declaration of Helsinki in the current Edinburgh version (2000).^10^ The diagnosis was confirmed by clinical, laboratory, and instrumental analysis according to the standard diagnostic protocols for AML^5^, ^11^ and formulated on the base of the WHO classification of tumors of hematopoietic and lymphoid tissues.^12^ Immunophenotyping and cytogenetic analysis were performed before the start of treatment as described in Reference 9. Demographic data including age, gender, date of presentation, time of relapse, and mortality were obtained from medical records or from institutional information databases. Patients received treatment in accordance with the standard clinical guidelines and recommendations.^13^, ^14^

AML patients were assigned into two groups: “primary” and “secondary.” Patients with newly diagnosed acute leukemia not receiving chemotherapy were attributed to “primary.” Patients with complicated hematological anamnesis and who had received chemotherapy previously for hematological disorders were attributed to “secondary.” Secondary leukemias are characterized by a poorer prognosis and a different therapeutic approach to primary leukemias, so they need to be analyzed separately.^15^, ^16^ Primary and secondary patients were also divided into groups according to the treatment they received as the first‐line therapy, in compliance with clinical recommendations. Patients who received induction therapy with anthracycline‐based regimens and those who received palliative therapy with protocols including low‐dose cytarabine due to severe concomitant pathology or serious disease complications were analyzed separately.

AML patients with HIV, hepatitis B and C, and tuberculosis were excluded from the study.

### 2.2 Collection of peripheral blood and bone marrow samples

Peripheral blood (PB) and/or bone marrow (BM) samples were obtained from leukemia patients (*n* = 37) at diagnosis before chemotherapy as described previously^9^ and were transported to the laboratory for in vitro studies within 3 hours of material collection.

### 2.3 Cell isolation and culture

Tumor cells were isolated from PB and/or BM of leukemia patients by centrifugation in lymphocyte separation medium (MP Biomedicals, USA), according to the manufacturer's instructions. The PB and/or BM samples were processed within 1‐3 hours following transportation to the laboratory. Cells were further cultured in the Iscove's Modified Dulbecco's Medium (IMDM) supplemented with 10% fetal bovine serum and a 1% solution of antibiotics and antimycotic (10 000 μg/mL streptomycin, 10 000 IU/mL penicillin, and 25 μg/mL amphotericin; ICN, Germany) at 37°C in a 5% CO_2_ humidified atmosphere for 12‐24 hours for adaptation to in vitro conditions.

### 2.4 Water‐soluble tetrazolium (WST)‐test

For drug sensitivity estimation, tumor cells isolated from PB and/or BM of leukemia patients were used. Cells were plated in 96‐well flat‐bottom plates at a density of 0.5 × 10^5^ to 2 × 10^5^ cells per well and were incubated in the presence of daunorubicin at concentrations of 0, 0.05, 0.1, 0.2, 0.4, 0.6, 1, and 2 μM, or cytarabine at concentrations 0, 0.001, 0.01, 0.2, 0.8, 4, 40, and 82 μM (both obtained from TEVA, Israel) for 72 hours at 37°C. Following incubation with chemotherapeutic drugs, 10 μL WST‐1 in a 0.5 mg/mL solution (Roche, Switzerland) was added to each well, and cells were incubated with WST‐1 for 3 hours at 37°C. Thereafter, the absorbance was measured spectrophotometrically using Multiscan RC (Labsystems, Finland) at 450 and 620 nm. The concentration of cytostatics that caused the death of 50% of tumor cells (IC_50_) was calculated as described in Reference 9.

Thus, the evaluation of the drug responsiveness of tumor cells in vitro takes no more than 120 hours from sample collection to the procurement of the WST‐test results.

### 2.5 Flow cytometry

Immunophenotyping of leukemia patients was performed by flow cytometry. BM and/or PB samples were incubated with VersaLyse Lysing Solution (A09777, Beckman Coulter, USA) at 37°C for 2 minutes. Then, leukemic cells were isolated by centrifugation at 400×*g* for 3 minutes and 30 seconds. The supernatant was removed, and the cells were washed and fixed with IOTest 3 Fixative Solution (A07800, Beckman Coulter) and incubated with monoclonal antibodies. The resulting samples were assayed using a Cytomics FC500 (Beckman Coulter) flow cytometer and CXP Software. At least 3 × 10^6^ cells were analyzed from each sample.

The expression of CD2, CD3, cCD3, CD4, CD5, CD7, CD10, CD11c, CD13, CD14, CD15, CD16, CD19, CD20, sCD22, CD23, CD25, CD26, CD30, CD33, CD34, CD38, CD43, CD45, CD56, CD61, CD64, CD65, CD71, cCD79a, CD103, CD117, CD138, HLA‐DR, TCR, FMC7, TdT, cMPO, Ki‐67, bcl‐2, and ZAP‐70 were analyzed.

### 2.6 Statistical analysis

Survival (number of patients = 127) was estimated using the Kaplan‐Meier method, and comparisons between groups were performed using the log‐rank test.

Correlation analysis (number of patients = 37) was performed using Spearman's rank order correlation coefficient (*r*_s_), which reflects the strength of the statistical relationship between the studied parameters: drug responsiveness, therapy response, and expression of immunological markers. All these variables were analyzed in addition to the treatment received. A value of *r*_s_ between 0.01 and 0.29 indicates of a weak positive correlation, between 0.30 and 0.69 a moderate positive correlation and between 0.70 and 1.0 a strong positive correlation. A value of *r*_s_ between −0.01 and −0.29 indicates a weak negative correlation, between −0.30 to −0.69—moderate negative correlation, and −0.70 to −1.0—strong negative correlation.

The data were analyzed using a Student's *t*‐test (unpaired, two‐tailed). Values of *P* ≤ .05 were considered statistically significant. All statistical analyses were performed with MS Excel, OriginPro 7.5, and Statistica 10.0.

## 3 RESULTS

### 3.1 Survival of AML patients

The survival rate of AML patients (*n* = 127) admitted to the Novosibirsk Hematology Center in the period from January 1, 2014 to December 31, 2018 was evaluated. The age and gender distributions of the patients are presented in Table S1.

**FIGURE 1:**
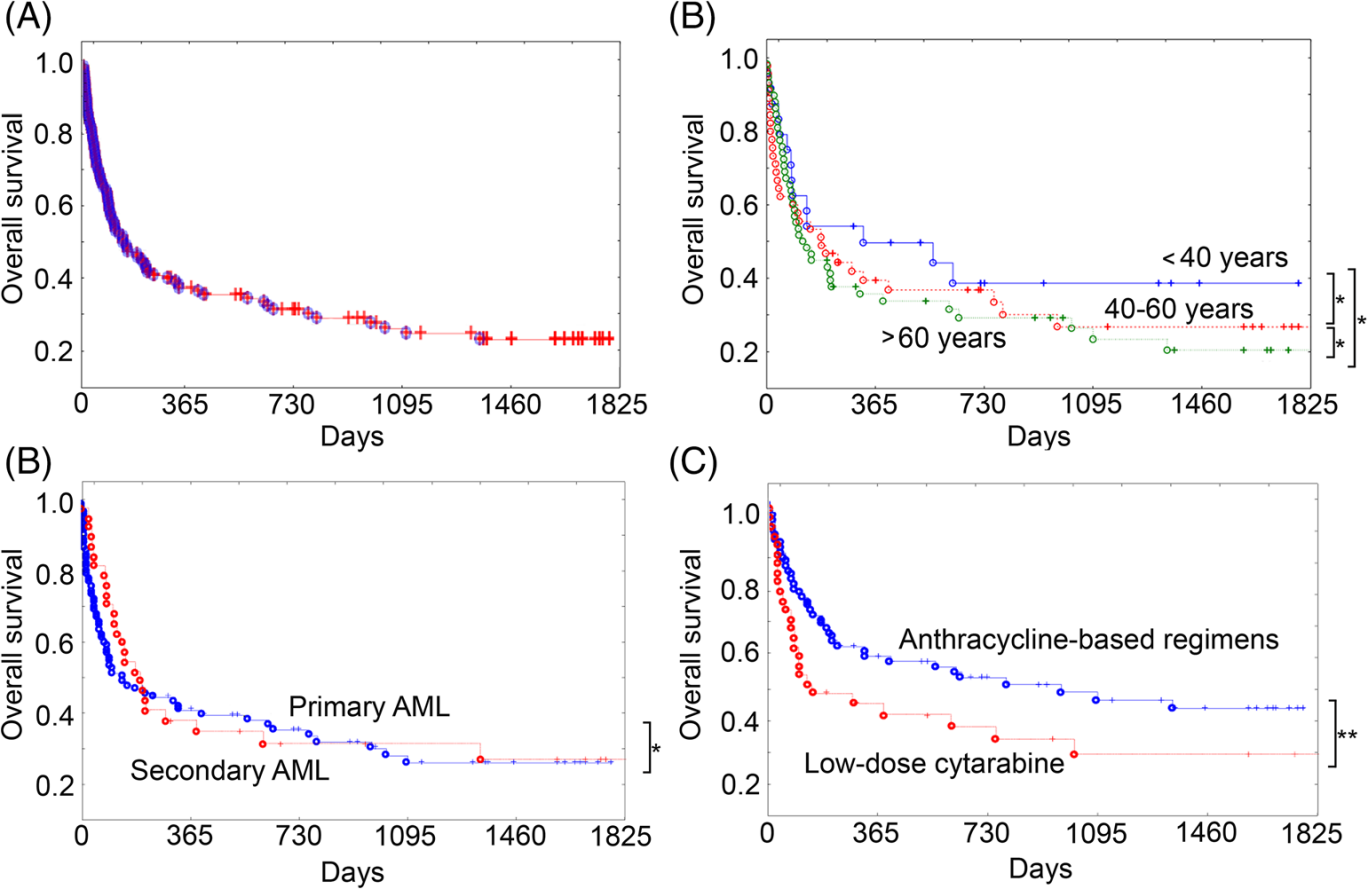
The Kaplan‐Meier survival curves for AML patients: total survival (A), survival depending on the patients' age (B), tumor status (primary or secondary) (C), and chemotherapy received (D). *P > .1, **P < .0001

The cumulative survival of patients with AML is demonstrated in Figure 1A: 3‐month survival was 60.6%, 6‐month survival 50.4%, 12‐month survival 40%, 3‐year survival 27.5%, and 5‐year survival 26%, in complete agreement with world statistics.^1^, ^2^

Next, we assessed the survival of AML patients according to age, tumor status (primary or secondary), and chemotherapy received as independent criteria in the prediction of survival.^17^, ^18^ The median survival rate in patients under the age of 40 years was 308.5 days, from 40 to 60 years 184 days, and older than 60 years 128.5 days (Figure 1B). In primary AML the median survival rate was 199 days, while in secondary AML it was 135 days (Figure 1C). Despite the differences between the groups were statistically insignificant, a tendency to decrease in the in patients older than 60 years and those with complicated hematological anamnesis (secondary AML) was observed, which is fully consistent with the published data.

As expected, patients who received induction chemotherapy with anthracycline‐based regimens demonstrated statistically significantly improved survival rates (median follow‐up: 292 days) compared with patients who received palliative chemotherapy with low‐dose cytarabine (median follow‐up: 107.5 days) (Figure 1D).

Then, we separately assessed the survival of patients who received anthracycline‐based therapy (*n* = 71), depending on the patient's age and the presence of primary or secondary AML. The median survival rate was 417, 242, and 214 days in patients under the 40 years, from 40 to 60 years and older than 60 years, respectively (Figure S1A). In primary AML patients who received anthracycline‐based regimens, the median survival rate was 367 days, while in secondary AML patients, it was 202 days (Figure S1B). However, differences between these patient groups were also not statistically significant, but with the tendency mentioned previously.

It is known that the prescribed treatment regimen is based on both patient‐related and disease‐related factors^5^; however, survival prediction in AML patients depends more on the antitumor therapy than on the patient's age and tumor status evaluated separately. Thus, only the chemotherapy received, which comprehensively reflects patient and disease conditions at diagnosis, defines the survival rate.

### 3.2 Responsiveness to chemotherapeutic drugs and therapy response of AML patients

We compared the drug responsiveness of tumor cells of leukemia patients at diagnosis with the therapy response depending on the treatment received. This part of the study included 37 patients with AML (22 primary and 15 secondary): 17 patients with primary AML received induction chemotherapy with anthracycline‐based regimens, and 5 patients with primary AML received palliative chemotherapy with low‐dose cytarabine; 8 patients with secondary AML received anthracycline‐based regimens, and 7 patients with secondary AML received low‐dose cytarabine (Table 1). It should be noted that such limited patients' cohorts may impose the restrictions in the extrapolation the results of our study into clinical practice and requires an additional enrollment of patients and further investigations, but despite this, important results were obtained.

**TABLE 1:** Sensitivity of tumor cells to chemotherapeutic drugs (WST‐test data), expression of immunological markers (flow cytometry data), cytogenetic abnormalities (karyotyping and FISH analysis data), treatment regimens, and therapy response of patients with acute myeloid leukemia with scales for correlation analysis (in parentheses)

Patient#	IC_50_, μM (scale)		Immunological markers	Cytogenetic abnormalities	Treatment regimens	Therapy response (scale)
	Daunorubicin	Cytarabine				
AML (primary)						
*Patients received anthracycline‐based regimens as a first‐line therapy*						
1	0.08 ± 0.01 (1)	60 ± 21.6 (3)	MPO, CD13, CD14, CD15, CD33, CD64, CD117, HLA‐DR	t(15;17)(q24;q21), P53 (17p13.1)	AIDA	Remission (1)
2	0.4 ± 0.05 (2)	—	—	Complex changes of karyotype	7 + 3; 7 + 3 + VP; 5 + 2	Remission(1)
3	>8 (3)	>82 (3)	—	Complex changes of karyotype	7 + 3; HAM; 6‐MP	Resistance (3)
4	0.2 ± 0.03 (1)	22.8 ± 3.7 (3)	MPO, CD7, CD11c, CD13, CD15, CD33, CD64, CD65, CD117, HLA‐DR	No	7 + 3; high‐dose cyclophosphamide; НАМ	Remission (1)
5	0.2 ± 0.05 (1)	0.6 ± 0.3 (1)	MPO, CD5, CD13, CD34, CD117, HLA‐DR, TdT	No	7 + 3; HiDAC; 6‐MP	Resistance (3)
6	0.2 ± 0.03 (1)	>82 (3)	MPO, CD11c, CD13, CD14, CD15, CD20, CD33, CD34, CD64, CD65, HLA‐DR	No	7 + 3; low‐dose cytarabine‐21	Remission (1)
7	0.01 ± 0.0001 (1)	0.3 ± 0.1 (1)	CD2, CD13, CD20, CD33, CD34, CD64, CD65, CD117	t(15;17)(q24;q21)	AIDA	Remission (1)
8	0.3 ± 0.05 (2)	64.4 ± 27 (3)	MPO, CD7, CD33, CD34, CD117, HLA‐DR	No	7 + 3	Relapse (2)
9	0.02 ± 0.001 (1)	2.3 ± 1.3 (2)	CD7, CD10, CD13, CD33, CD34, CD71, CD117	No	7 + 3; low dose cytarabine‐14; 5 + 2; intermediate‐dose cytarabine‐7	Relapse (2)
10	0.02 ± 0.002 (1)	0.6 ± 0.4 (1)	MPO, CD13, CD14, CD15, CD33, CD64, CD117	t(15;17)(q24;q21)	AIDA	Remission (1)
11	0.2 ± 0.01 (1)	0.9 ± 0.2 (1)	MPO, CD13, CD14, CD15, CD33, CD34, CD64, CD65, CD117	No	7 + 3	Relapse (2)
12	—	0.7 ± 0.2 (1)	—	t(15;17)(q24;q21)	7 + 3 + vesanoid; 6‐MP	Remission (1)
13	>3.5 (3)	>40.8 (3)	—	No	7 + 3; HiDAC; low‐dose cytarabine‐21; 6‐MP	Resistance (3)
14	1.3 ± 0.4 (3)	>82 (3)	MPO, CD10, CD13, CD14, CD15, CD33, CD64, CD65	Trisomy 8	7 + 3; low‐dose cytarabine‐21	Resistance (3)
15	>2 (3)	>82 (3)	MPO, CD11c, CD15, CD20, CD25, CD33, CD56, CD64, CD71, CD117, HLA‐DR, TdT	Trisomy 8, deletion 11	7 + 3	Resistance (3)
16	0.3 ± 0.02 (2)	71.5 ± 21 (3)	MPO, CD33, CD34, CD117, HLA‐DR	Deletion 5, TAS2R1(5p15.31), RELN(q22), TES(q31), P53(17p13.1)	Low‐dose cytarabine‐21; 5 + 2	Resistance (3)
17	—	22 ± 11.2 (3)	—	No	7 + 3	Remission (1)
*Patients received low‐dose cytarabine as a first‐line therapy*						
18	>2 (3)	>82 (3)	MPO, CD13, CD33, CD34, CD117, HLA‐DR	No	Low‐dose cytarabine‐21	Resistance (3)
19	0.2 ± 0.02 (1)	2.9 ± 0.7(2)	MPO, CD15, CD20, CD33, CD64, CD65, CD117, HLA‐DR	MLL(11q23.3)	Low‐dose cytarabine‐21 + 6‐MP	Remission (1)
20	>8 (3)	>82 (3)	—	No	Low dose cytarabine‐14; 6‐MP	Resistance (3)
21	0.01 ± 0.002 (1)	0.9 ± 0.4 (1)	—	No	Low‐dose cytarabine‐21	Resistance (3)
22	0.4 ± 0.3 (2)	16.9 ± 5.5 (3)	—	Trisomy 9, deletion 11	Low‐dose cytarabine‐21; 6‐MP	Resistance (3)
AML (secondary)						
*Patients received anthracycline‐based regimens as a first‐line therapy*						
23	>2 (3)	>82 (3)	MPO, CD2, CD3, CD4, CD5, CD7, CD13, CD33, CD34, CD117, HLA‐DR	No	5 + 2; 7 + 3; HAM; intermediate‐dose cytarabine‐7	Resistance (3)
24	>2 (3)	>82 (3)	CD13, CD34, CD117, HLA‐DR	i(17)(q11)	Low‐dose cytarabine‐14; 5 + 2	Resistance (3)
25	0.2 ± 0.035 (1)	1.1 ± 0.3 (1)	MPO, CD7, CD11c, CD13, CD34, CD117, HLA‐DR	Deletion 5, RELN(q22), TES(q31)	Low‐dose cytarabine‐28; 5 + 2; 6‐MP	Resistance (3)
26	0.35 ± 0.08 (2)	3 ± 0.6 (2)	—	No	5 + 2; 7 + 3; low‐dose cytarabine‐14	relapse (2)
27	>8 (3)	>82 (3)	—	Complex changes of karyotype	5 + 2; low‐dose cytarabine‐28; 7 + 3; VAD; 6‐MP	Resistance (3)
28	—	>82 (3)	—	No	5 + 2	Resistance (3)
29	—	3.6 ± 0.2 (2)	MPO, CD11c, CD13, CD14, CD15, CD33, CD64, CD65, CD71, HLA‐DR	MLL(11q23.3)	7 + 3; AIDA; intermediate‐dose cytarabine‐7; 6‐MP	Relapse (2)
30	>2 (3)	>82 (3)	MPO, CD7, CD13, CD15, CD33, CD34, CD64, CD65, CD117, HLA‐DR	Trisomy 8	5 + 2; low‐dose cytarabine‐21; 6‐MP	Resistance (3)
*Patients received low‐dose cytarabine as a first‐line therapy*						
31	>2 (3)	>82 (3)	MPO, CD2, CD13, CD15, CD16, CD33, CD34, CD64, CD65, CD117	No	Low‐dose cytarabine‐21; low‐dose cytarabine‐14	Resistance (3)
32	0.2 ± 0.02 (1)	29 ± 5 (3)	CD13, CD33, CD34, CD56, CD64, CD117, HLA‐DR	RELN (q22), TES(q31)	Low‐dose cytarabine‐21	Resistance (3)
33	4.4 ± 1.5 (3)	>82 (3)	—	t(9;22)(q34;q11)	Low‐dose cytarabine‐21 + dasatinib	Remission (1)
34	0.2 ± 0.01 (1)	2 ± 0.5 (2)	CD13, CD33, CD34, CD65, CD117, HLA‐DR	No	Low‐dose cytarabine‐21	Relapse (2)
35	0.5 ± 0.1 (2)	>82 (3)	MPO, CD10, CD33, CD34, CD56, CD117, HLA‐DR	RELN (q22), TES(q31)	Low‐dose cytarabine‐21; 6‐MP	Resistance (3)
36	0.2 ± 0.01 (1)	13.6 ± 3.3 (3)	—	Deletion 5, P53(17p13.1)	Low‐dose cytarabine‐28; 6‐MP	Resistance (3)
37	—	>161 (3)	—	No	Low‐dose cytarabine‐14	Relapse (2)

*Note: AIDA*: ATRA 45 mg/m^2^ per os (30 days) + idarubicin 12 mg/m^2^ intravenously (2, 4, 6, 8 days). *7 + 3*: cytarabine 100‐200 mg/m^2^ intravenously (1–7 days) + daunorubicin 45‐60 mg/m^2^ intravenously (1–3 days). *7 + 3 + VP*: cytarabine 100‐200 mg/m^2^ intravenously (1‐7 days) + daunorubicin 45‐60 mg/m^2^ intravenously (1‐3 days) + etoposide 120 mg/m^2^ intravenously (17‐21 days). *5 + 2*: cytarabine 100‐200 mg/m^2^ intravenously once a day (1‐5 days) + daunorubicin 45‐60 mg/m^2^ intravenously (1, 2 days). *6‐MP*: 6‐mercaptopurine 50 mg/m^2^ intravenously. *Low‐dose cytarabine* −*14*, −*21*, −*28*: 10 mg/m^2^ subcutaneously twice a day (during 14, 21 or 28 days). *Intermediate‐dose cytarabine‐7*: 600 mg/m^2^ twice a day (during 7 days). *HAM*: high‐dose cytarabine 3 g/m^2^ intravenously twice a day (1‐3 days) + mitoxantrone 10 mg/m^2^ intravenously (3‐5 days). *HiDAC*: high‐dose cytarabine 3 g/m^2^ intravenously twice a day (1, 3, 5 days). *High‐dose cyclophosphamide*: 7 g/m^2^ intravenously + G‐CSF 5 mg/kg intravenously. *VAD*: vincristine 0.4 mg/m^2^ intravenously (1‐4 days) + adriablastine 10 mg/m^2^ intravenously (1‐4 days) + dexamethasone 40 mg/m^2^ per os (1‐4 days, 11‐14 days).

The responsiveness of tumor cells to daunorubicin and cytarabine was estimated using the WST test as the preferred method for cell viability assessment.^19^ Sensitivity to daunorubicin was evaluated, since this drug refers to anthracyclines mostly used for induction therapy in AML patients. Sensitivity to cytarabine was evaluated, since it is used for palliative therapy in AML patients with severe comorbidities and disease complications (infectious, hemorrhagic, etc.).^20^ The obtained IC_50_ values for the studied drugs for each leukemia patient, as well as immunological markers, treatment regimens and therapy responses, are listed in Table 1. As seen from the presented data, patients with primary AML were predominately sensitive to daunorubicin (10 patients—high sensitivity, 4 patients—moderate sensitivity, 6 patients—resistance, and 2 patients—not detected) and resistant to cytarabine (6 patients—high sensitivity, 2 patients—moderate sensitivity, 13 patients—resistance, and 1 patient—not detected) (Table 1). Patients with secondary AML mainly demonstrated resistance both to daunorubicin (4 patients—high sensitivity, 2 patients—moderate sensitivity, 6 patients—resistance, and 3 patients—not detected) and cytarabine (1 patient—high sensitivity, 3 patients—moderate sensitivity, and 11 patients—resistance) (Table 1).

When the therapy response was evaluated, it was found that patients with primary AML receiving induction chemotherapy mostly had a good response to treatment and achieved remission, but there were also patients who were initially resistant to treatment (8 patients—remission, 3 patients—relapse, and 6 patients—resistance) (Table 1). Patients with primary AML receiving palliative chemotherapy mostly demonstrated resistance (1 patient—remission and 4 patients—resistance) (Table 1). Patients with secondary AML had a poor response to therapy regardless of the treatment received (1 patient—remission, 4 patients—relapse, and 11 patients—resistance) (Table 1).

Next, we developed appropriate scales for subsequent correlation analysis (see Table S2) as described in Reference 9, and all patients were scaled according to the responsiveness of their tumor cells to chemotherapeutic drugs, therapy response, and expression of immunological markers (Table 1).

### 3.3 Correlation analysis

To perform retrospective correlation analysis, patients with primary and secondary AML were divided according to the treatment protocols received: patients treated with anthracycline‐based induction chemotherapy (cohort 1 and cohort 3 for primary and secondary AML, respectively) and patients treated with palliative chemotherapy by low‐dose cytarabine (cohort 2 and cohort 4 for primary and secondary AML, respectively). These cohorts of patients were analyzed separately to determine the correlations between the sensitivity of tumor cells to chemotherapeutic drugs and the therapy response depending on the treatment received. The data obtained are summarized in Table 2.

**TABLE 2:** Correlation coefficients reflecting the relationships between the drug sensitivity, expression of immunological markers in tumor cells and therapy response of AML patients

	Primary AML						Secondary AML					
	Induction chemotherapy (cohort 1)			Palliative chemotherapy (cohort 2)			Induction chemotherapy (cohort 3)			Palliative chemotherapy (cohort 4)		
	IC_50_ daunorubicin	IC_50_ cytarabine	Therapy response	IC_50_ daunorubicin	IC_50_ cytarabine	Therapy response	IC_50_ daunorubicin	IC_50_ cytarabine	Therapy response	IC_50_ daunorubicin	IC_50_ cytarabine	Therapy response
Therapy response	0.68*	0.12	1.0	0.54	−0.33	1.0	0.56	0.54	1.0	−0.54	—	1.0
**CD‐markers**												
**Immature markers**												
HLA‐DR	0.14	0.23	0.12	—	—	—	—	—	—	—	—	—
TdT	0.24	−0.58	0.50	—	—	—	—	—	—	—	—	—
CD34	−0.42	−0.50	—	0.89	0.27	0.83	0.14	−0.07	0.07	0.42	—	—
**Lymphoid markers**												
**T‐cell**												
CD2	0.53	0.19	0.38	—	—	—	0.41	0.31	0.20	0.56	—	—
CD3	0.53	0.14	0.45	—	—	—	0.58	0.56	0.40	—	—	—
CD5	−0.26	−0.76*	0.57	—	—	—	0.50	0.33	0.33	—	—	—
CD7	0.40	0.37	0.08	—	—	—	0.76	0.65	0.75	—	—	—
CD11c	0.33	1.0	—	—	—	—	—	—	—	—	—	—
**B‐cell**												
CD20	0.09	0.25	−0.38	—	—	—	—	—	—	—	—	—
CD56	0.67	0.22	—	−0.87	—	−1.0	—	—	—	−0.75	—	—
**Myeloid markers**												
CD13	−0.90*	−0.55	−0.46	—	—	—	—	—	—	0.46	—	—
CD14	−0.45	0.39	−0.44	—	—	—	—	−0.19	−0.79	—	—	—
CD15	—	0.57	−0.76*	−0.87	—	−1.0	—	0.50	—	−0.08	—	—
CD16	—	—	—	—	—	—	—	−0.58	−0.82	0.54	—	—
CD33	0.48	0.99*	−0.05	—	—	—	0.27	0.45	0.28	0.08	—	−0.54
CD64	—	0.72	−0.71	−0.87	—	−0.50	0.50	—	−0.24	0.18	—	—
CD65	−0.40	0.39	−0.53	—	—	—	0.50	0.82	−0.54	0.56	—	—
CD117	0.43	−0.33	0.52	0.54	1.0	−0.33	—	−0.02	0.50	—	—	—

*Note*: Red color indicates strong positive correlation (0.70 ≤ *r*_s_ ≤ 1.00), blue color indicates moderate positive correlation (0.30 ≤ *r*_s_ ≤ 0.69), and green color indicates weak positive correlation (0.01 ≤ *r*_s_ ≤ 0.29), — indicates absence of any correlations. * indicates statistically significant differences at *P* ≤ 0.05.

In cohort 1, the therapy response exhibited a moderately positive correlation with the sensitivity of tumor cells to daunorubicin (*r* = 0.68) and a weak correlation with sensitivity to cytarabine (*r* = 0.12) (Table 2). Patients in cohort 2 demonstrated a moderate correlation between the therapy response and sensitivity to daunorubicin (*r* = 0.54) as well as a negative correlation between the therapy response and sensitivity to cytarabine (*r* = −0.33) (Table 2). In cohort 3, moderate positive correlations between the therapy response and the sensitivity of tumor cells to daunorubicin (*r* = 0.56) and cytarabine (*r* = 0.54) were found (Table 2). Patients in cohort 4 demonstrated no correlations between the therapy response and cell responsiveness to cytotoxic drugs (Table 2).

Analysis of the correlations between the responsiveness of tumor cells to chemotherapeutic drugs and the expression of immunological markers that have unfavorable predictive value for leukemia patients (markers of immaturity and an aberrant immunophenotype)^21^ shows that in patients of cohort 1 and cohort 2 (both belong to primary AML), correlations between the resistance of leukemic cells to cytostatics and positive expression of immature markers were predominantly weak or absent (Table 2). On evaluation of aberrant immunophenotype markers in cohort 1, T‐cell and B‐cell lymphoid markers had predominantly moderate correlations with sensitivity to daunorubicin (CD2, CD3, CD7, CD11c, CD56) and weak correlations with sensitivity to cytarabine (CD2, CD3, CD7, CD11c, CD20, CD56) (Table 2). In cohort 2, no correlations between drug sensitivity and aberrant markers were detected.

When the relationships with immature markers for secondary AML were evaluated, only correlations between sensitivity to daunorubicin and expression of CD34 in cohort 3 (weak) and cohort 4 (moderate) were found (Table 2). As for markers of an aberrant immunophenotype, in cohort 3, moderate and strong correlations between T‐cell markers and sensitivity to daunorubicin (CD2, CD3, CD5, CD7) and cytarabine (CD2, CD3, CD5, CD7) were found (Table 2). In cohort 4, a moderate correlation between sensitivity to daunorubicin and CD2 expression only was detected (Table 2).

Correlation analysis confirms our assumption that the therapy response of leukemia patients correlates with the drug responsiveness of tumor cells estimated in vitro. Patients with high sensitivity to daunorubicin retrospectively demonstrated good response to anthracycline‐based therapy. Patients resistant to daunorubicin, but received it as part of induction chemotherapy according to the standard clinical recommendations, did not respond to treatment. Regarding cytarabine, the correlations are not so clear: patients sensitive to cytarabine, but resistant to daunorubicin and received low‐dose cytarabine due to comorbidity and complicated hematological anamnesis, did not respond well to the palliative chemotherapy. Thus, the responsiveness of tumor cells to daunorubicin can reflect the total sensitivity to chemotherapy. Correlations of drug resistance with unfavorable prognostic immunological markers are the additional confirmation of the validity of our approach.

## 4 DISCUSSION

Although leukemia accounts only for 4% of all cancers, this heterogeneous group of hematological disorders remains the sixth most lethal malignancy in the world.^22^ AML is the most common leukemia in adults with approximately 50% 1‐year survival in elderly patients.^23^, ^24^ Our study showed that the survival rates of AML patients receiving standard chemotherapy are consistent with published statistical data. A comparison of lifespan depending on age, tumor status (primary or secondary), and therapy received revealed that the treatment regimen is the defining factor in patient survival. This may be related to the fact that prognostic factors evaluated separately (such as age and tumor status) have a lesser predictive impact on survival than a factor taking into account several variables, such as prescribed treatment.^5^

Approximately 80% success in AML treatment is achieved with standard chemotherapy.^18^, ^25^ The standard induction chemotherapy for AML patients still remains the anthracycline‐based regimen by the 7 + 3 protocol including cytarabine 7 days and daunorubicin 3 days.^20^, ^26^ When induction therapy is not possible due to the severe comorbidity and complicated hematological anamnesis of the patient, palliative therapy by low‐dose cytarabine is used.^18^, ^27^

One of the main predictors of unfavorable prognosis for leukemia patients is the resistance of tumor cells to cytotoxic drugs, which can be both initially found at diagnosis and acquired as a result of chemotherapy.^28^ The presence of an aberrant immunophenotype, as well as a mixed phenotype of leukemia, is an additional marker of poor prognosis.^29^, ^30^ When the therapeutic response of leukemia patients was evaluated retrospectively as a function of the drug responsiveness of tumor cells and the treatment received, some important correlations were revealed. Patients with high sensitivity of tumor cells to cytostatics in vitro who were receiving these drugs as a part of their treatment had a good response to therapy. Conversely, patients whose tumor cells exhibited resistance to a number of cytostatics in vitro and who received them within standard clinical protocols had a poor response to treatment. Associations of drug resistance with adverse factors such as immaturity and an aberrant immunophenotype were also found.

Current strategies for drug responsiveness assessment include combinations of gene expression markers, the mutational status of leukemic cells and standard clinical and laboratory variables.^31^, ^32^ However, techniques based on genetic profiling are time consuming. Our approach allows the determination of a treatment strategy and the commencement of proper chemotherapy within 5 days of diagnosis. Moreover, once drug resistance can be detected in patients' tumor cells, the relevant approaches may be taken to overcome it.

The common approaches to overcome drug resistance in clinical practice are the administration of cytotoxic drugs not previously used by the particular patient^33^; a shift to high doses of previously used cytotoxic drugs^34^; continuous exposure to low doses of chemotherapeutic drugs^35^; induction of tumor cell differentiation^36^; use of drugs based on herbal extracts and their derivatives which have a wide range of biological activities^37^, ^38^ and may inhibit the binding of cytostatics with P‐glycoprotein.^39^ All these approaches can be used both independently and in combination with standard chemotherapy.

Thus, using a retrospective approach, we proved our conception that an evaluation of the initial responsiveness of tumor cells to chemotherapeutic drugs in vitro can be useful in predicting the therapy response of leukemia patients and can be applied to newly diagnosed AML to assign the most effective antitumor treatment.

## 5 CONCLUSIONS

We demonstrated that the survival of leukemia patients predominately depends on the treatment received, and the response of leukemia patients to chemotherapy correlates with the drug responsiveness of tumor cells estimated in vitro at diagnosis. Thereby, evaluation of the initial sensitivity/resistance of tumor cells to chemotherapeutic drugs may be useful in predicting the patient's response to planned chemotherapy and may serve as a substantial basis for modification of standard treatment protocols to overcome drug resistance in leukemia patients.

## AUTHOR CONTRIBUTIONS

**Maria Kolesnikova:** Data curation; formal analysis; investigation; validation. **Aleksandra Sen'kova:** Data curation; formal analysis; investigation; methodology; writing‐original draft. **Tatiana Pospelova:** Conceptualization; methodology; project administration; supervision. **Marina Zenkova:** Conceptualization; funding acquisition; methodology; project administration; resources; supervision; writing‐review & editing.

## CONFLICT OF INTEREST

The authors declare that no conflict of interest exists.

## ETHICAL STATEMENT

All patients were enrolled in the study by signing the informed consent and with ethical approval from the Institutional Review Board of Novosibirsk State Medical University (No. 80/2015) in accordance with the ethical principles of the Declaration of Helsinki in the current Edinburgh version (2000).

## Supporting information

**FIGURE S1** Survival of AML patients who received anthracycline‐base regimens as first‐line therapy depending on patients' age (A) and primary or secondary AML (B). *P > .1.
**TABLE S1** Age and gender distribution of AML patients (*n* = 127).
**TABLE S2** Scales for assessment the drug responsiveness of tumor cells, the expression of immunological markers and the response to therapy in AML patients.

## Data Availability

The data that support the findings of this study are available on request from the corresponding author. The data are not publicly available due to privacy or ethical restrictions.
