# Supplementary material for: Drug responsiveness of leukemic cells detected in vitro at diagnosis correlates with therapy response and survival in patients with acute myeloid leukemia
Source: Cancer Rep (Hoboken). 2021 Mar 6;4(4):e1362. doi: 10.1002/cnr2.1362 (PMC8388166; doi:10.1002/cnr2.1362)
Supplement: Supplementary file 1 — FIGURE S1 Survival of AML patients who received anthracycline‐base regimens as first‐line therapy depending on patients' age (A) and primary or secondary AML (B). *P > .1. TABLE S1 Age and gender distribution of AML patients (n = 127). TABLE S2 Scales for assessment the drug responsiveness of tumor cells, the expression of immunological markers and the response to therapy in AML patients. [file CNR2-4-e1362-s001.docx]

**Table S1.** Age and gender distribution of AML patients (n = 127).

| Age | Male | | Female | |
| --- | --- | --- | --- | --- |
|  | Abs. | % | Abs. | % |
| < 40 years | 10 | 17.2 | 14 | 20.3 |
| 40-60 years | 23 | 39.7 | 22 | 31.9 |
| > 60 years | 25 | 43.1 | 33 | 47.8 |
| Total | 58 | 100 | 69 | 100 |


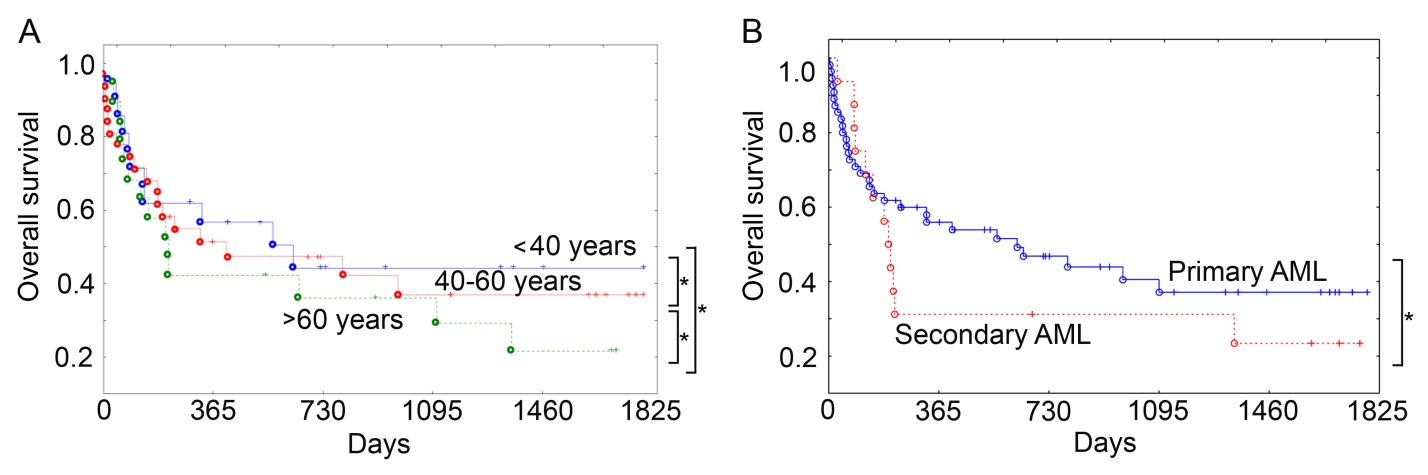


**Figure S1.** Survival of AML patients who received anthracycline-base regimens as first-line therapy depending on patients’ age (A) and primary or secondary AML (B). **p* > 0.1.

**Table S2.** Scales for assessment the drug responsiveness of tumour cells, the expression of immunological markers and the response to therapy in AML patients.

|  | **Scale 1** | **Scale 2** | **Scale 3** |
| --- | --- | --- | --- |
| *Drug sensitivity (WST-test)* | *High sensitivity* | *Moderate sensitivity* | *Low sensitivity (resistance)* |
| Daunorubicin (IC_50_, μM) | 0 – 0.25 | 0.25 – 0.5 | > 0.5 |
| Cytarabine (IC_50_, μM) | 0 – 1.5 | 1.5 – 8 | > 8 |
| *Therapy response* | *Remission* | *Relapse with subsequent remission* | *Relapse with subsequent resistance or initial resistance* |
| Number of blasts in the BM after 1-2 courses of chemotherapy, % | < 5 | 5 – 20 | > 20 |
| *Expression of immunological markers* | *Negative* | *Positive* | - |
| Number of blasts expressed the antigen, % | < 20% for surface markers, < 10% for cytoplasmic markers | > 20% for surface markers, > 10% for cytoplasmic markers | - |
